# Surface Functionalization of a Silica-Based Bioactive Glass with Compounds from *Rosa canina* Bud Extracts

**DOI:** 10.1021/acsbiomaterials.0c01170

**Published:** 2020-12-11

**Authors:** Giulia Ferlenda, Martina Cazzola, Sara Ferraris, Andrea Cochis, Ajay Kumar, Enrico Prenesti, Silvia Spriano, Enrica Vernè

**Affiliations:** † Politecnico di Torino, Department of Applied Science and Technology, Institute of Materials Physics and Engineering, Corso Duca degli Abruzzi, 24, 10129, Torino, Italy; ‡ Department of Health Sciences, Center for Translational Research on Autoimmune and Allergic Diseases - CAAD, University of Piemonte Orientale UPO, c.so Trieste 15/A, 28100, Novara, Italy; § Department of Chemistry, University of Torino, Via Pietro Giuria 7, 10125, Torino, Italy

**Keywords:** surface functionalization, bioactive glasses, polyphenols, bud extracts, *Rosa canina*

## Abstract

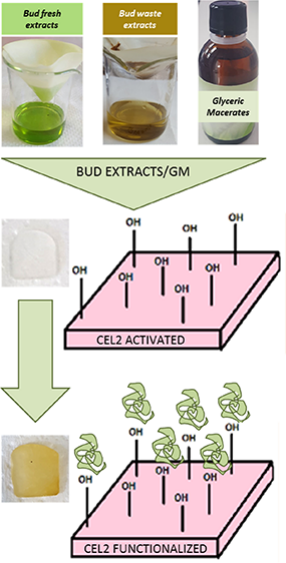

Bud extracts are a new category of vegetal products, which are used in gemmotherapy. These products are liquid preparation sources of bioactive molecules (phytochemicals) and are used in medicine as health-promoting agents. *Rosa canina* is a medicinal plant belonging to the family *Rosaceae*. The *R. canina* bud extracts, in particular, possess anti-inflammatory and antioxidant activities due to the presence of flavonoids and other phenolic compounds. The combination of *R. canina* bud extracts with biomaterials can be promising for obtaining multifunctional materials carrying both inorganic and biological properties. In this work, a protocol of functionalization has been properly designed, for the first time in the literature, in order to graft various bud extracts of *R. canina* to a silica-based bioactive glass (CEL2). The Folin–Ciocalteu method was used to determine the redox capacity of total polyphenols in the extracts and on functionalized solid samples. X-ray photoelectron spectroscopy analysis and fluorescence microscopy were employed to investigate the presence of phenol substances on the material surface. Bioactivity (in terms of ability of inducing hydroxyapatite precipitation) has been investigated by soaking the samples, with or without functionalization, in simulated body fluid. The presence of the polyphenols from bud extracts not only preserved glass bioactivity but even enhanced it. In particular, the solution obtained from the byproducts of primary extraction in glycerol macerate showed the best performances. Moreover, the presence and antioxidant activity of bud extract compounds on the material surface after grafting demonstrate the possibility of combining the glass inorganic bioactivity with the biomolecule-specific properties, making possible a local action at the implant site. The promising results reported in this work pave the way for the realization of new multifunctional materials with a green approach.

## 1 Introduction

In the last few years, the use of herbal medicinal products increased strongly due to their potential health benefits and low toxicity. According to the World Health Organization (WHO), about 80% of the world population are using products based on medicinal herbs, especially in developing countries.^1,2^

One type of phytoderivate product is bud extracts, obtained exclusively from fresh buds, sprouts, young leaves, and other meristematic tissues, which are macerated in a mixture of water, ethanol, and glycerol, the result consisting of concentrated solutions of bioactive phytoingredients. Buds are rich in bioactive compounds such as vitamins, enzymes, proteins, amino acids, nucleic acids, growth factors, micropolypeptides, plant hormones, and cytokines. In addition, gemmo-derivatives contain beneficial substances that can no longer be found in the adult plant, such as gibberellin, auxin, or cytokines.^3−5^ The use of buds makes it possible to obtain a more active medication than remedies prepared from the whole plant. The official procedure for bud preparation is detailed in the monograph “Homeopathic preparations” published in the 8th edition of the French Pharmacopoeia and the subsequent edition.^6^ Commercial liquid preparations derived from *Rosa caninaL.* (Dog Rose) buds or young sprouts are one of the most used plants in traditional folk medicine for their high phenolic contents. Almost all of the studies available in the literature have focused on evaluating rose hip and seed extracts, while to date, scientific papers on the bud extracts have been minimal or are completely^7−12^ Several compounds from rose hip extracts have been reported to display in vitro anti-inflammatory and antioxidant activities.^7−9^ Orodan et al. reported that the proanthocyanidins and flavonoids contained in *R. canina* fruits possess radical scavenging properties. The rose hip extract activities were higher than other reference antioxidants (such as 2-mercaptoethane sulfonate (mesna) and *N*-acetylcysteine) against HClO and H_2_O_2._^10^ Chrubasik et al. reported a beneficial effect of rose hip powder in the treatment of osteoarthritis.^11^ Schwager et al. demonstrated that rose hip powder has enhanced in vitro anti-inflammatory and chondroprotective properties in human peripheral blood leukocytes and primary chondrocytes.^12^ Rose hips are known to have a very high vitamin C content, far exceeding that in citrus fruits.^13−16^ In addition, rose hips contain other vitamins and mineral components, carotenoids, tocopherols, flavonoids, fruit hydroxy acids, tannins, pectin, sugars, amino acids, and essential oils rich in volatile substances.^16^ Recent studies revealed that the *R. caninaL*. extracts were effective for the inhibition of growth and biofilm formation of methicillin-resistant *Staphylococcus aureus* (MRSA).^17,18^

Surface functionalization is a useful and versatile procedure to realize multifunctional materials, combining the properties of both substrates and grafted molecules. It is currently possible to modify biomaterial surfaces for implants with chemical and biological functionalization for the realization of drug delivery systems, by grafting molecules with a covalent bonding, or by simple adsorption.^19,20^ The peculiar properties of the grafted molecules can be combined with those of the substrate for a local action at the site of implant.

Bioactive glasses are a particular class of biomaterials of interest for bone contact applications due to their ability to form chemical bonds with bone and stimulate its growth and regeneration. One of the main applications of bioactive glasses is bone implants, and it is therefore necessary to control the physical, chemical, and biochemical properties of implant surfaces in order to improve tissue integration. Some studies have been developed in the last few years concerning the opportunity to bind natural molecules to bioactive glasses in order to couple the properties of inorganic materials with those of phytochemicals.^21−27^ Gallic acid, a natural molecule present in many plants, has been combined with a bioactive glass as a model molecule for polyphenols and in order to take advantage of its antioxidant, anti-allergic, antibacterial, anticarcinogenic, and anti-mutagenic properties.^22,23^ Polyphenols extracted from grape skins and green tea leaves have been grafted to the surface of a bioactive glass without the use of any synthetic spacer.^21,23,28^

Despite the increasing interest in the application of bud extracts in homeopathic treatments, in the scientific literature, there are no studies that combine bud extracts with biomaterials. The main purpose of the present work is to study the possibility of grafting different bud extracts of *R. canina* to a bioactive glass surface, in different grafting conditions. Due to the absence in the literature of valuable procedures to promote the interaction between bud extracts and solid surfaces, in this work, we propose for the first time the study and the appropriate design of a protocol of functionalization, to graft the active principles of the buds to a bioactive glass surface in a stable and reproducible way. The glass surface after functionalization has been characterized in order to assess the effectiveness of the grafting procedure and its eventual influence on the glass bioactivity (in terms of ability to induce hydroxyapatite precipitation). The grafting of *R. canina* bud extracts to the bioactive glass surface allows a local action of these molecules at the site of implant in a synergistic way with the bioactive glass itself for a multifunctional activity, aimed at a more physiological healing (bone stimulation, modulation of the inflammatory response, control of infection). Bioactive glasses functionalized with natural bud extracts can be promising materials for bone contact applications in critical situations, such as bone loss due to cancer resection or infections. Particularly, the use of *R. canina* bud extracts can be of interest for bone contact application due to the abovementioned properties. Moreover, in the perspective of a circular economy, a further novelty of the present work is represented by the utilization of biomolecules from natural sources, exploiting the byproducts of bud extracts (bud post-maceration) still rich in active ingredients^29^ and transforming a waste product into a resource.

## 2 Materials and Methods

### 2.1 Sample Preparation

In the present research work, a silica-based bioactive glass (CEL2) developed and characterized in previous works^30−32^ was used as the substrate (in bulk and powder form) for the grafting of various bud extracts. This glass composition has been chosen continuing from the previous experience of the research group on surface functionalization of the same substrate with different biomolecules.^31,32^ The glass was produced by the traditional melt and quenching route, and its molar composition is 45% SiO_2_, 3%P_2_O_5_, 26% CaO, 7% MgO, 15% Na_2_O, and 4% K_2_O. After melting of the precursors (SiO_2_, Ca_3_(PO_4_)_2_, CaCO_3_, C_4_H_2_Mg_5_O_14_·5H_2_O, Na_2_CO_3_, and K_2_CO_3_, >99%, Sigma Aldrich) in a platinum crucible at 1500 °C for 1 h, the melted glass was poured in water to obtain a frit or poured on a brass plate to obtain bars. The glass bars were annealed at 500 °C for 13 h in order to release residual stresses,^21,33^ cut in slices of 2 mm-thick (Struers Accutom 5), and polished with SiC abrasive paper (120–4000 grit). Glass slices with homogeneous surfaces and a total area of 124.12 ± 12.16 mm^2^ were obtained. The frit was milled and sieved up to a grain size lower than 20 μm. Each powder sample used for the tests was composed of 100 mg of CEL2 powders.

### 2.2 Phenol Compound Handling

The biomolecules used for the functionalization of the bioactive glass were bud extracts of *R. canina* (Table 1).

**Table 1:** Acronyms of Samples/Solutions and Description of the Functionalization Procedures with Bud Extracts of *R. canina*^a^

sample acronym	sample description
MG ROSA	glyceric macerate of *R. canina*
MG ROSA WEG	glyceric macerate of *R. canina* diluted to 1/10 in water/ethanol/glycerol
MG ROSA W	glyceric macerate of *R. canina* diluted to 1/10 in water
BUDS ROSA	*R. canina* fresh bud extract
BY-PRODUCT ROSA	*R. canina* glyceric macerate byproduct extract
CEL2	CEL2 washed (acetone and water)
CEL2 + MG ROSA	CEL2 functionalized with glyceric macerate of *R. canina*
CEL2 + MG ROSA WEG	CEL2 functionalized with glyceric macerate of *R. canina* diluted to 1/10 in water/ethanol/glycerol
CEL2 + MG ROSA W	CEL2 functionalized with glyceric macerate of *R. canina* diluted to 1/10 in water
CEL2 + BUDS ROSA	CEL2 functionalized with *R. canina* fresh bud extract
CEL2 + BYPRODUCT ROSA	CEL2 functionalized with *R. canina* glyceric macerate byproduct extract

The glyceric macerate of *R. canina* (MG ROSA) was provided by GEALPHARMA (Bricherasio, Torino, Italy), a small company manufacturing glyceric macerates and mother tinctures in Piedmont.

Glyceric macerate (henceforth known as MG) was prepared according to the European Pharmacopea 8th edition, following the procedure deriving from the French Pharmacopea^6^ with some changes. Briefly, buds were left to macerate in a mixture of 50 wt % water, 20 wt % ethanol, and 30 wt % glycerol, with a solid/liquid ratio of 1:15. After 3 months of maceration, the suspension was filtered, and the residue was pressed. The percolate was added to the filtrate, and the obtained solution was stored in stainless steel containers and then transferred in glass vessels (MG ROSA) or diluted as explained in Table 1 and in Section 2.3. (MG ROSA WEG and MG ROSA W).

*R. canina* fresh bud extracts were obtained from buds collected in the north west of Italy (Prali, Piedmont). Conventional solvent extraction was performed in a water/ethanol mixture (20:80 volume ratio) with a solid/liquid ratio of 1:20. The extraction was made in a thermostatic bath, at 60 °C for 60 min under shaking (120 rpm). The extraction solution was separated from the buds using a filter and put into an incubator at 37 °C until the total ethanol evaporation. Finally, the extracts were picked and suspended in double-distilled water and freeze-dried (BUDS ROSA).

Another type of extraction was made using the bud post-maceration that was used for a *R. canina* glyceric macerate by-product extract. The extraction was done with almost the same procedure followed by the fresh buds, omitting the freeze drying step, since the residual glycerol contained in the buds after the first maceration made freeze drying not possible.

### 2.3 Glass Surface Activation

In order to functionalize a surface, the presence of reactive functional groups on it is essential, such as free hydroxyl groups.^22,23^ The method of exposing of the −OH groups has already been optimized in previous works^32,33^ and, briefly, consists of a washing step in acetone first in an ultrasonic bath for 5 min, to remove the surface contaminants, and then three additional washing steps for 5 min in double-distilled water in order to expose the −OH groups. The samples with the surface activated will be named glass-washed from now on.

### 2.4 Surface Functionalization

Five solutions of bud extracts were prepared for glass functionalization: 1.0 mg/mL bud rosa lyophilized in double-distilled water (BUDS ROSA), glyceric macerate of *R. canina* (MG ROSA), MG ROSA diluted to 1/10 in a mixture of 50 wt % water, 20 wt % ethanol, and 30 wt % glycerol (MG ROSA WEG), MG ROSA diluted to 1/10 in water (MG ROSA A), and 10 mg/mL buds post-maceration (BY-PRODUCT ROSA).

The glass slices were put into a holder coated with aluminum foil to prevent the UV light degradation of phenol, covered with 5 mL of one of the five solutions previously prepared, and incubated for 3 h at 37 °C following a protocol developed from previous works.^22,23^ After that time, the slices were washed twice in double-distilled water and dried at room temperature. Three samples functionalized with each solution were prepared for each test.

The samples grafted with the bud extract were named CEL2 + MG ROSA, CEL2 + MG ROSA WEG, CEL2 + MG ROSA A, CEL2 + BUDS ROSA, and CEL2+ BY-PRODUCT ROSA (Table 1).

### 2.5 Photometric Analysis

The total phenolic content and redox activity of the bud extracts were measured using the Folin–Ciocalteu method.^30^ The solution (2 mL) was mixed with 6 mL of double-distilled water and with 0.5 mL of Folin–Ciocalteu reagent (Folin–Ciocalteu phenol reagent, Sigma Aldrich). After 3 min, 1.5 mL of 20 wt % Na_2_CO_3_ solution was added, and after 2 h of reaction, the photometric reading was performed. The absorbance was measured at λ = 760 nm using a Beckman DU 64 UV–VIS spectrophotometer. A standard curve of calibration was prepared by using different concentrations of gallic acid (0.0025, 0.005, 0.01, 0.02, 0.03, and 0.04 mg/mL) as described in ref (1). The total phenolic content was expressed as the ratio mg gallic acid/mL functionalization solution (GA equivalent, GA = gallic acid).

To quantify the redox capacity of the polyphenols grafted on the surface, a modified version of the Folin–Ciocalteu test was performed: the glass slices functionalized was put into a holder covered with 8 mL of water, 0.5 mL of Folin–Ciocalteu reagent, and 1.5 mL of 20% (p/V) Na_2_CO_3_ solution.^34,35^

All determinations were performed in triplicate.

### 2.6 Fluorescence Microscopy Observations

In order to verify the presence and the distribution of the biomolecules grafted on the surface and their distribution, functionalized glasses were observed in different areas by fluorescence microscopy (Leica DM5500 B, Leica Microsystems, IL, USA) exploiting the natural autofluorescence of polyphenols.^36^

### 2.7 XPS Analysis

To evaluate the presence of the polyphenols on the surface, X-ray photoelectron spectroscopy analysis (XPS, PHI 5000 VERSAPROBE, PHYSICAL ELECTRONICS) of bulk samples was made. Both functionalized and non-functionalized samples were analyzed.

Survey spectra were acquired in order to determine the chemical composition of the surfaces, while the high-resolution spectra of the most significant elements (C and O) were recorded in order to investigate the chemical state of elements and determine the presence of chemical groups characteristic of the polyphenols from bud extracts.

### 2.8 Apatite-Forming Ability Tests

To investigate the bioactivity of the new biomaterial in terms of the apatite-forming ability of glass before and after functionalization, the glass bulk samples were soaked in simulated body fluid (SBF).^37,38^ The powder samples (100 mg), one for each type, were put in a bottle coated with aluminum foil, to avoid polyphenol photodegradation, and covered with 25 mL of SBF according to previous work.^22,23^ All the samples were incubated at 37 °C for up to 14 days, the SBF was refreshed every 3 days, and the pH was measured in order to evaluate the variation due to the ionic release from the glass. After the soaking in SBF, the samples were dried at room temperature. The samples were analyzed after 3, 7, and 14 days by means of FTIR (Nicolet iS50 FTIR Spectrometer) on pellets of the samples with 198 mg of KBr and 2 mg of glass powder.

### 2.9 Statistical Analysis of Data

Experiments were performed in triplicate. Results were statistically compared by the SPSS software (v25, IBM) using the one-way ANOVA test and Tukey’s post-hoc analysis. Results were considered significant for *p* < 0.05.

## 3 Results and Discussion

### 3.1 Macroscopic Observations and pH Measurements

**Figure 1:**
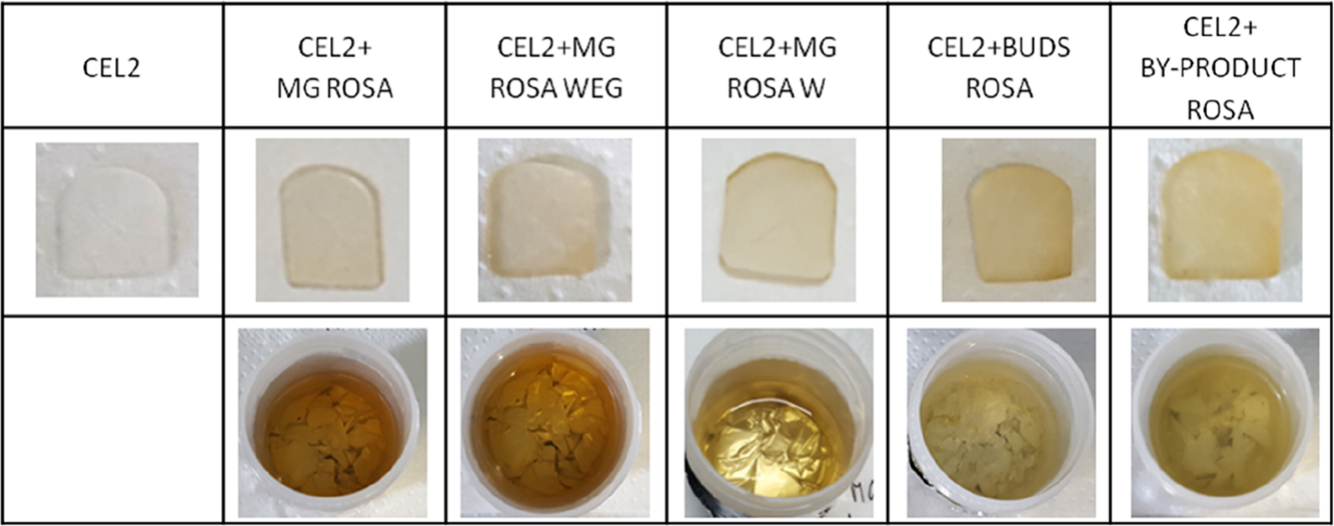
Macroscopic observation of glasses before and after functionalization (upper panel) and solutions (lower panel) before soaking samples in simulated Body Fluid (SBF). The yellow/orange color of the glasses after functionalization confirmed that biomolecules were successfully grafted onto the specimens’ surface.

Figure 1 shows the CEL2 bulk samples functionalized and solutions before the functionalization process. It is clearly visible that the surface of CEL2 changes color from colorless to yellow-orange after functionalization due to biomolecule grafting.

**Figure 2:**
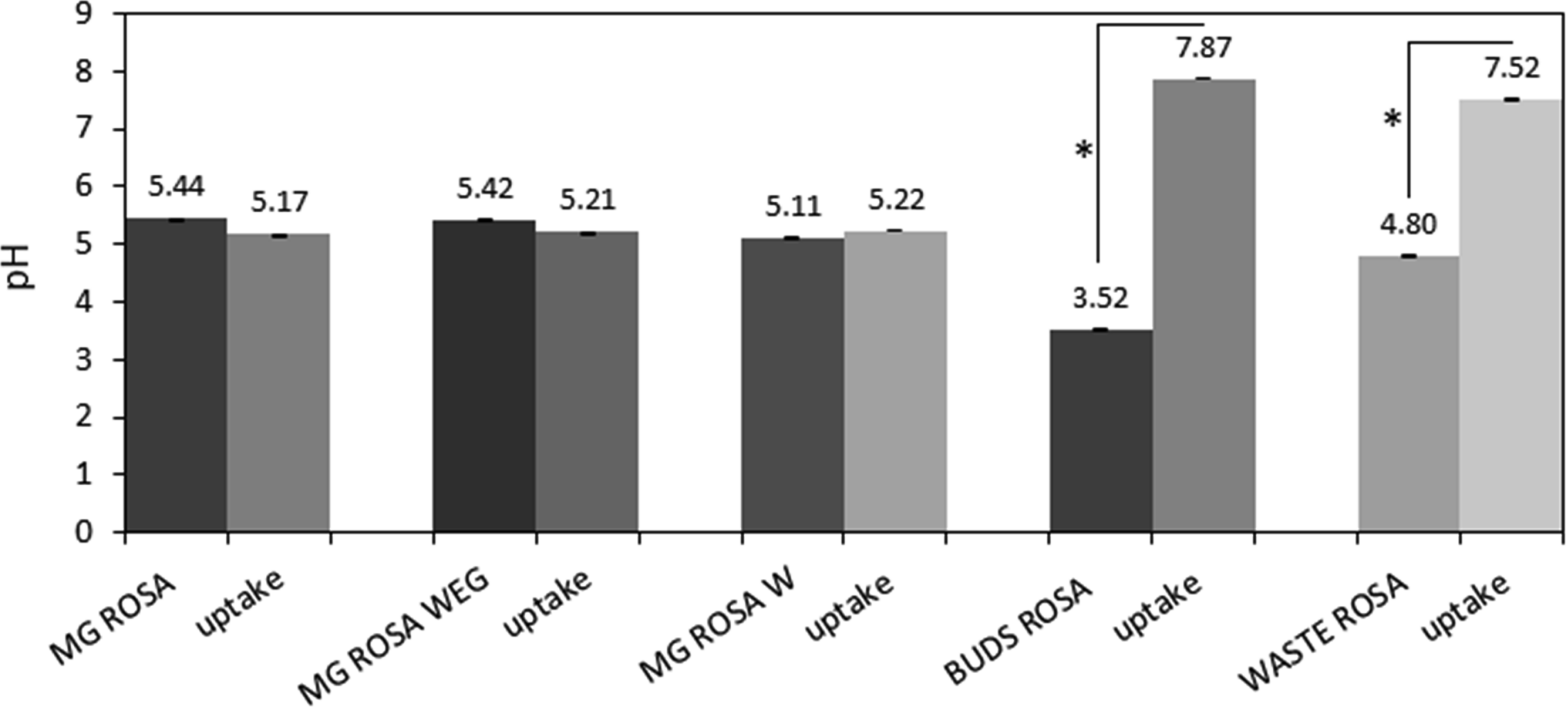
Evaluation of the pH value of *R. canina* solutions before and after (uptake) 3 h of soaking of different glass bulks. The use of BUDS ROSA and WASTE ROSA determined a significant pH toning toward basic values in comparison with the starting values (*p* < 0.05, indicated by *).

Then, in order to investigate the effect of pH, the pH value of the *R. canina* solution was measured before and after soaking CEL2 for 3 h (the term “uptake” concerns the functionalization solutions after soaking) and the results are shown in Figure 2.

All five utilized solutions were characterized by an acidic pH, but the pH changes after functionalization depending on the presence or absence of glycerol. The ion release of bioactive glasses in the solution medium BUDS ROSA and BYPRODUCT ROSA (solutions without glycerol) causes a significant increase in pH up to a basic value in comparison to the starting values (*p* < 0.05, indicated by *); the initial pH values of the functionalization solutions are 3.52 and 4.80, respectively, while they become 7.87 and 7.52 after the soaking (3 h of functionalization time).

It must be underlined as already performed for gallic acid, tea, and grape polyphenols^21−23^ that, in the present setup, the glasses were soaked in unbuffered solutions. MG ROSA, MG ROSA WEG, and MG ROSA W contain glycerol, and it is in these three solutions that no particular pH changes were recorded. The literature lacks references concerning surface functionalization with solutions containing glycerol, so the reason for the unchanged pH in the soaking solution could be only hypothesized and related to a barrier effect opposed by the glycerol adsorbed in the liquid phase on the glass surface that hinders ion exchange.

### 3.2 Folin–Ciocalteu Test

The Folin–Ciocalteu test was performed on the samples functionalized and on the solutions before and after the procedure of functionalization, in order to measure the quantity of polyphenols and their redox activity.

This test is not only a quantitative measurement of polyphenols in the solutions or on the surfaces after grafting, but it also reveals whether the molecules are still active (redox reactivity) after coupling with bioactive glasses.

**Figure 3:**
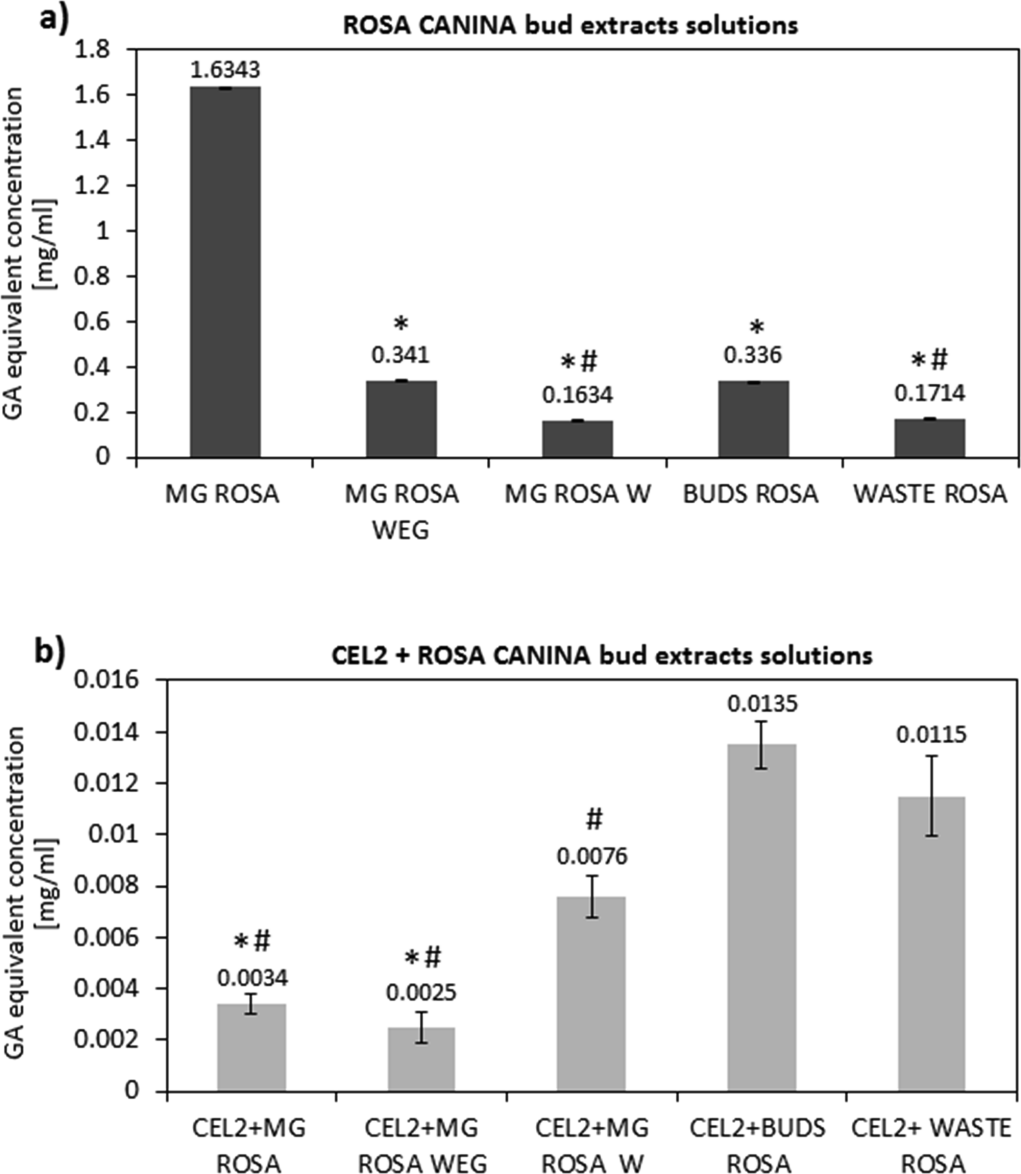
Phenol amount evaluation by the Folin–Ciocalteu assay. When extract solutions were considered (a), a significant reduction of phenol amount was observed for the diluted solutions in comparison with pure MG ROSA (*p* < 0.05, indicated by *); moreover, the phenol amount on MG ROSA W and WASTE ROSA resulted significantly lower than MG ROSA WEG and BUDS ROSA (*p* < 0.05, indicated by #). Looking at the functionalized glasses (b), it was noticed that the use of glycerol lowered the amount of grafted biomolecules, thus making CEL2 + MG ROSA and CEL2 + MG ROSA WEG the worst specimens (*p* < 0.05 vs other specimens, indicated by *). The use of water (CEL2 + MG ROSA W) ameliorated the amount of phenol that was anyway significantly lower than CEL2 + BUDS ROSA and CEL2 + WASTE ROSA (*p* < 0.05, indicated by #).

Figure 3a reports the concentrations of polyphenols in the solutions used for grafting expressed in the unit of GA equivalent. It can be observed that the phenol concentration in MG ROSA is significantly higher (1.634 mg/mL GA equivalent) than that of the other solutions (*p* < 0.05, indicated by *). Moreover, by diluting MG ROSA with a mixture of 50 wt % water, 20 wt % ethanol, and 30 wt % glycerol and pure water, the concentration of polyphenols is significantly lowered, as expected. Accordingly, a significant difference in terms of phenol concentration was observed by comparing MG ROSA WEG to MG ROSA W and BUDS ROSA (*p* < 0.05, indicated by #); also, WASTE ROSA showed a significantly lower biomolecule amount in comparison with BUDS ROSA (*p* < 0.05, indicated by #).

To check for interferences, a measurement was made on samples treated in ethanol/glycerol/water, which gave zero as a response: it can be concluded that no interference from glycerol is observed in this measurement.

Functionalized bulk samples were investigated after functionalization with *R. canina* extracts (Figure 3b). It can be noted that the amount of bud extracts grafted on the glass strongly depends on the medium used. Dilution of MG in water increases the concentration of polyphenols on the surface probably due to the weaker presence of glycerol. Glycerol acting like a barrier seems to reduce the reactivity of glass, as observed with the pH measurement, as reported in Figure 2, and it can also act as a physical barrier that isolates the surface of the samples from grafting and inhibits ion exchange between the glass surface and solution. Thus, CEL2 + MG ROSA and CEL2 + MG ROSA WEG showed a significantly lower phenol amount in comparison with the other combinations (*p* < 0.05, indicated by *); similarly, CEL2 + MG ROSA W showed to be significantly less functionalized by the biomolecules in comparison with both CEL2 + BUDS ROSA and CEL2 + WASTE ROSA (*p* < 0.05, indicated by #).

### 3.3 Fluorescence Microscopy Observations

**Figure 4:**
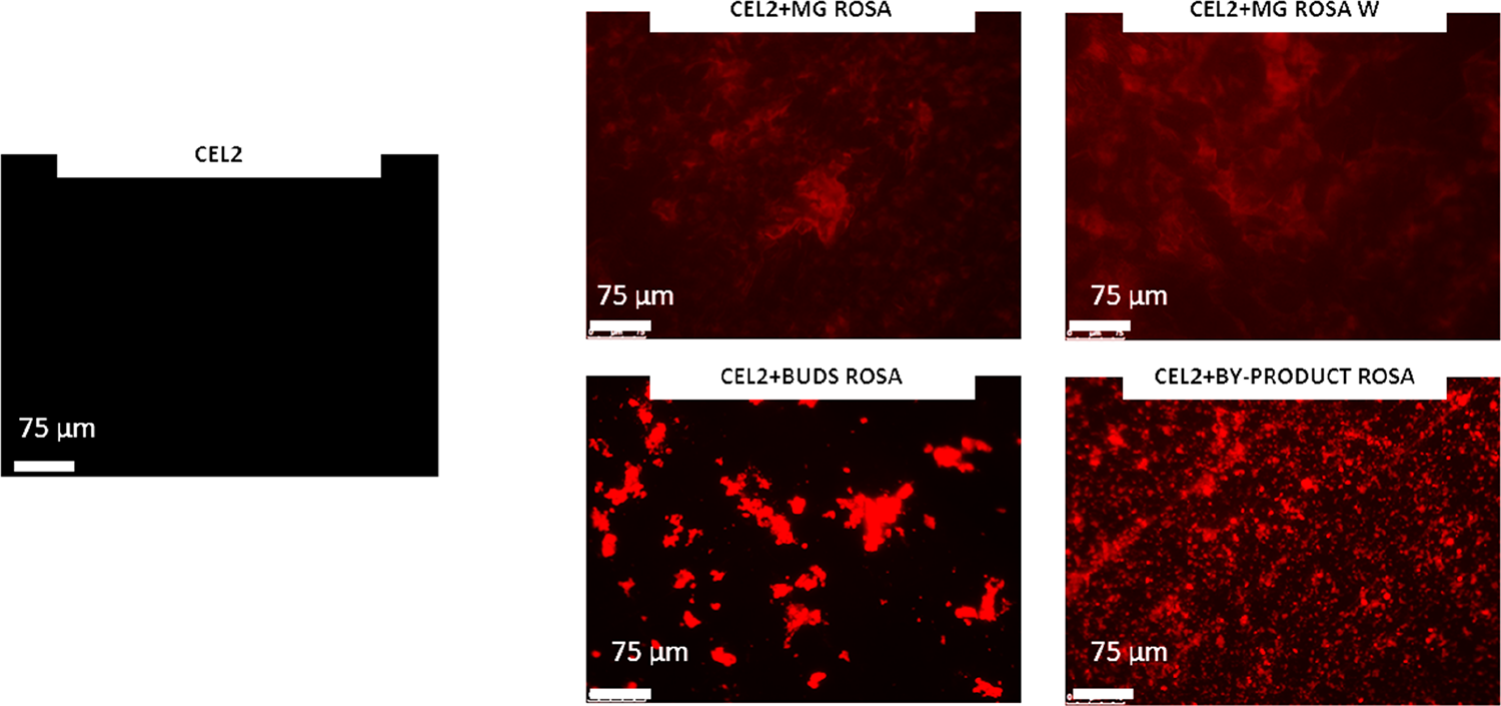
Fluorescence images of CEL2, CEL2 + MG ROSA, CEL2 + MG ROSA W, CEL2 + BUDS ROSA, CEL2 + BY-PRODUCT ROSA. The detected red signals are due to the polyphenols auto-signal thus confirming the correct grafting of the biomolecules to the glasses’ surface.

Representative fluorescence images of control (CEL2) and functionalized samples (CEL2 + MG ROSA, CEL2 + MG ROSA W, CEL2 + BUDS ROSA, and CEL2 + BYPRODUCT ROSA) are reported in Figure 4. Fluorescence was applied to visually check for the correct polyphenols grafting onto the glasses’ surface as they produce a strong autofluorescence signal as we previously showed for red grape skin and green tea leaves.^28^ The control CEL2 does not show any signal as expected, thus confirming the lack of a bulk signal due to the glasses’ composition. Conversely, functionalized glasses showed an obvious fluorescence signal due to the biomolecules that grafted onto the surface. These images highlighted the success of the procedure of functionalization and the presence of a homogeneous layer of polyphenols on the surface of the glass with some brighter spots on the samples CEL2 + BUDS ROSE and CEL2 + BYPRODUCT ROSA due to a local stronger presence of grafted polyphenols.

### 3.4 XPS Analysis

XPS analysis was employed to characterize the chemical composition and bonds on the surface of bare CEL2 bulk samples and those functionalized with *R. canina*.

Table 2 reports the atomic percentages of C, O, and Si detected on the surface of bioactive glasses before (CEL2) and after polyphenol grafting. It can be observed that a certain amount of carbon contaminants is observable on the CEL2 surface, as reported in the literature for reactive surfaces.^32,39−41^

**Table 2:** Atomic Percentages of C, O, and Si (at %) from XPS Survey Analyses Detected on Samples

element	CEL2	CEL2 + MG ROSA	CEL2 + MG ROSA W	CEL2 + BUDS ROSA	CEL2+ BYPRODUCT ROSA
C	37.5	57.2	53.7	53.7	55.9
O	44.0	34.2	36.4	37.1	36.1
Si	13.7				
other	4.8	8.6	9.9	9.2	8.0

The most important information given by XPS analysis is the absence of Si on all the functionalized samples, which suggests the presence of a layer of natural molecules (thicker than the XPS penetration depth, at about 4 to 5 nm) that covers the glass. A significant increase (about 20%) in the carbon content after functionalization suggests the presence of organic molecules on the surface.

**Figure 5:**
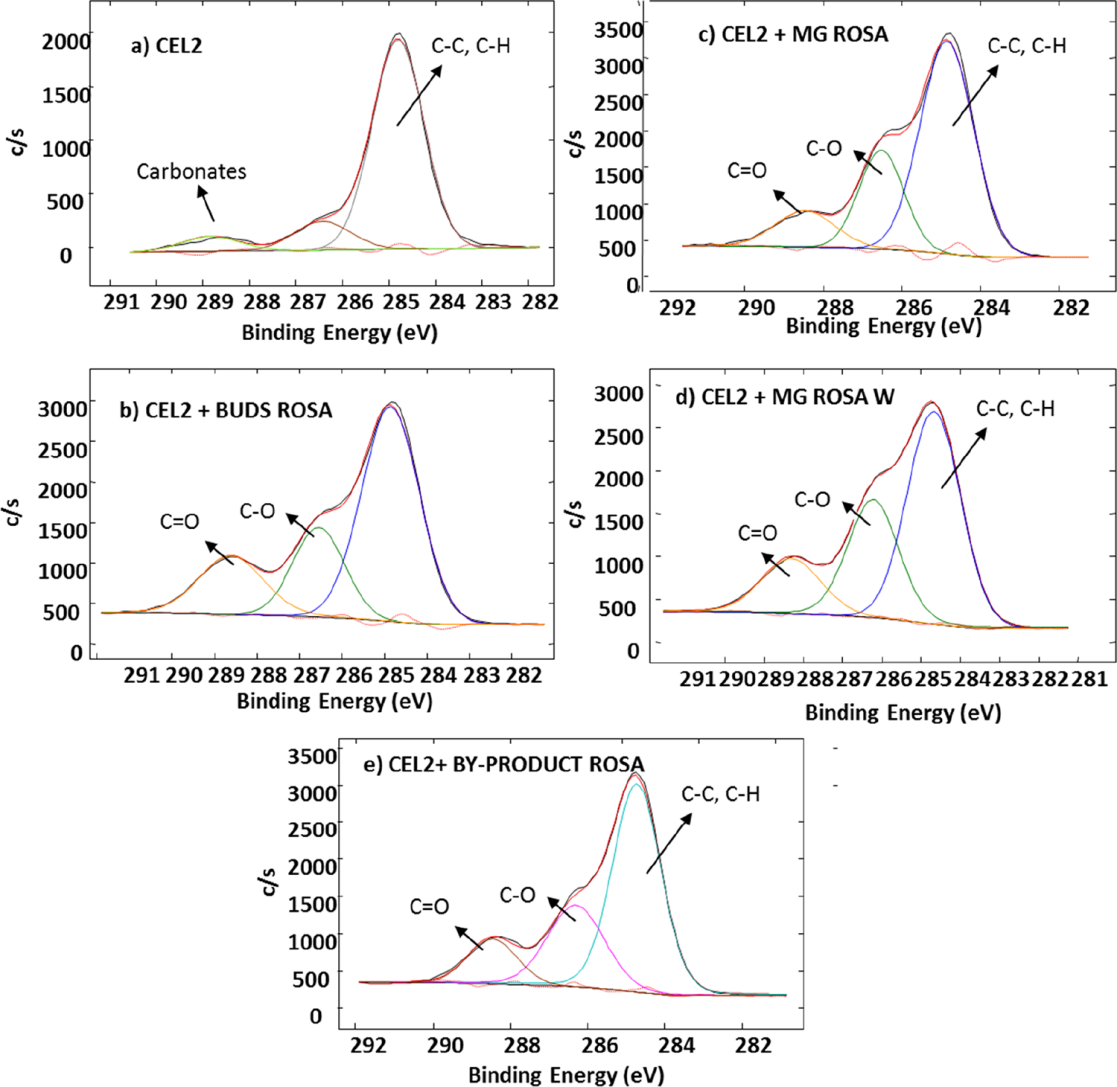
XPS high-resolution spectra of the carbon region of CEL2, CEL2 + MG ROSA, CEL2 + MG ROSA W, CEL2 + BUDS ROSA, and CEL2 + BYPRODUCT ROSA.

**Figure 6:**
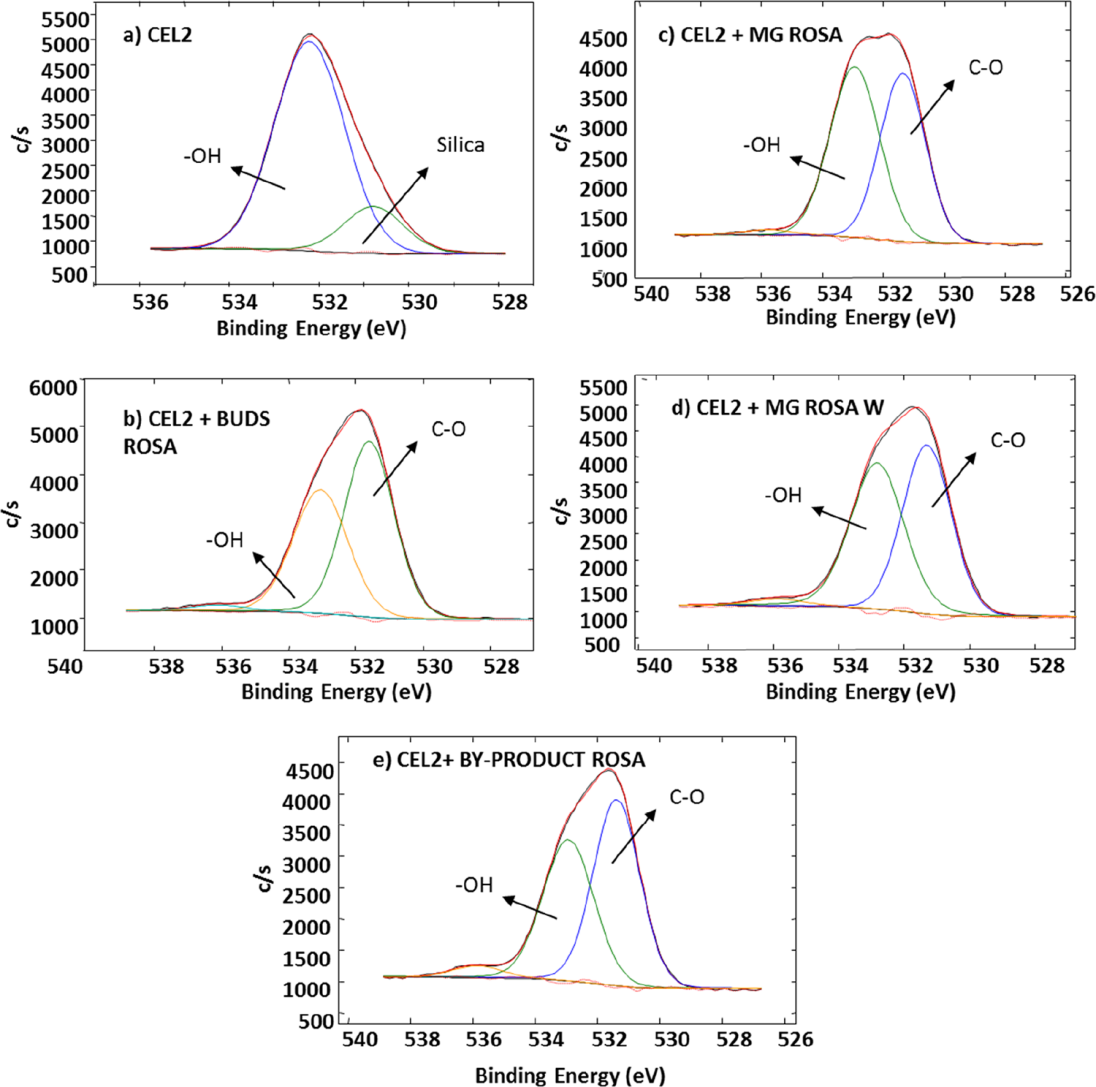
XPS high-resolution spectra of the oxygen region of CEL2, CEL2 + MG ROSA, CEL2 + MG ROSA W, CEL2 + BUDS ROSA, and CEL2 + BYPRODUCT ROSA.

In order to identify the chemical groups exposed on the surfaces, the detailed analyses of carbon and oxygen regions have been performed and reported in Figures 5 and 6.

Figure 5 shows the high-resolution XPS spectra of the carbon region of CEL2 bulk samples before and after functionalization.

A notable signal at 284.79 eV was detected on the surface of washed CEL2, which can be attributed to unavoidable hydrocarbon contaminations on reactive surfaces as mentioned in the literature for XPS analysis of reactive materials.^42−44^ The signal at about 289.25 eV can be assigned to carbonates, usually observed on the surface of bioactive glasses as the contaminant.^22,23^ The signal attributed to carbonates disappears after *R. canina* functionalization, as previously observed by the authors after functionalization with gallic acid and grape and tea polyphenols.^21−23^ Moreover, two other peaks at 286.54 and 288.60 eV were observed. These peaks can be attributed to C–O and C=O bonds according to the literature^22,23^ and they are characteristic of polyphenols, which confirms the presence of these molecules on the surface. On the contrary, the signal at 284.79 eV still persists and it can be attributed both to surface contamination and to C–C and C–H bonds in the polyphenol molecules. The increased intesity of these last peaks can be correlated to the increase of the atomic percentage of carbon content on the surface functionalized with polyphenols.

Figure 6 shows the high-resolution spectra of the oxygen region. The first spectrum is related to washed CEL2 and underlines the presence of the characteristic signal for silica at 530.80 eV and hydroxyls at 532.22 eV as reported in the literature related to this glass after surface activation.^37^

The signal attributed to Si–O bonds disappears on functionalized samples, in accordance with the absence of the Si signal in the survey spectra. On the other hand, the signal attributed to the −OH groups persists and showed to have shifted to higher energies compared to that of the washed glass. This shift can be associated with the presence of aromatic OH typical of phenols.^42^ Moreover, a signal at about 531.6 eV appears on functionalized samples and can be attributed to C=O bonds, present in polyphenols, in accordance with the results obtained in the carbon region. The functional groups of glycerol are mainly C–H, C–O, and OH: as a consequence, it is not possible to discriminate them from those of polyphenols and individuate eventual surface bonding of glycerol. Since the sample simply treated with the ethanol, glycerol, and water mixture was not responsive to the Folin–Ciocalteu test, as reported in section 3.2., the grafting of glycerol to the substrates can be considered negligible.

### 3.5 Apatite-Forming Ability Tests

Powder glass samples were soaked in SBF up to 14 days to investigate the bioactivity in terms of hydroxyapatite precipitation. The pH was checked in order to evaluate the variation due to the ionic release from the glass, and it ranged between 7.40 and 8.18.

**Figure 7:**
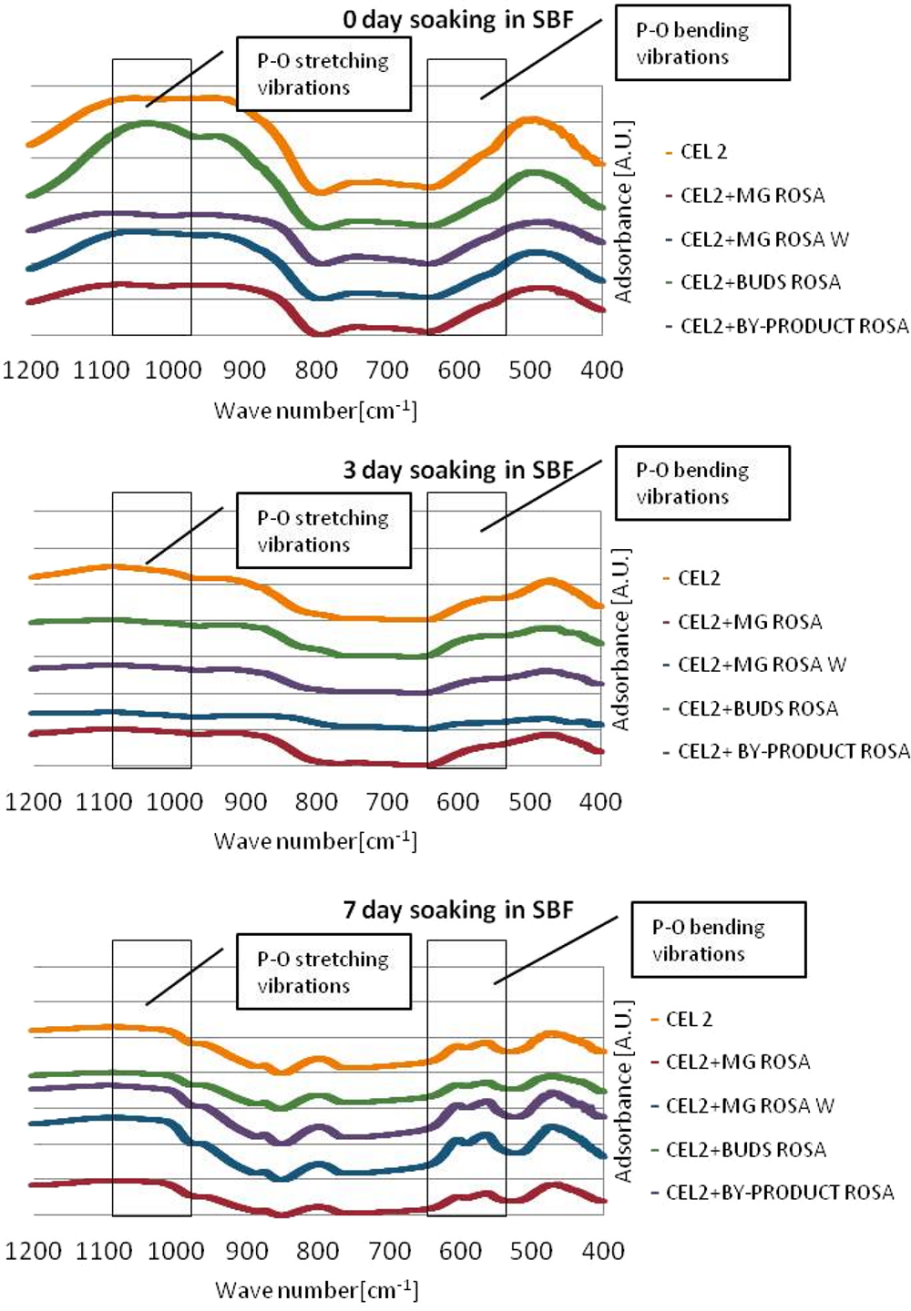
IR spectra of glass powder before (CEL2) and after functionalization with polyphenols after different times of soaking in SBF. Results demonstrated that the presence of grafted polyphenols did not inhibit the glasses’ bioactivity as the typical peaks of phosphates in hydroxyapatite (around 600 and 560 cm^–1^) were detected 3 and 7 days after soaking in SBF.

The powder glasses were analyzed after 3, 7, and 14 days by means of FTIR and IR spectroscopy, and the results are reported in Figure 7.

The presence of hydroxyapatite on pellet samples is shown by a double peak around 600 and 560 cm^–1^. This double peak is correlated with the bending vibration of P–O bonds. It is evident that functionalization does not inhibit glass bioactivity of pure CEL2. These results are in accordance with the ones previously observed by the authors for surface functionalization of CEL2 with gallic acid and polyphenols from grapes and tea.^21−23^ This point is extremely important because it confirms the possibility of coupling the typical properties of the substrate (e.g., bioactivity for the bioactive glass) with those of the grafted molecules. Considering that glycerol seems to reduce the ion exchange of bioactive glasses (as reported in the investigation of pH variations in the functionalization media), the results of bioactivity tests support the hypothesis, previously reported in the XPS discussion, that glycerol does not remain grafted on the glass surface after functionalization, while it is adsorbed on the glass surface in the liquid phase.

## 4 Conclusions

In this work, a protocol of functionalization of bioactive glasses with the phytoextract of *R. canina* buds was developed. A silica-based bioactive glass named CEL2 was exploited as a substrate, and different extracts from buds were used for functionalization. The measurements of pH on the solutions of functionalization before and after soaking of the samples showed that the presence of glycerol prevents the basification of the solutions, suggesting a lower reactivity of the surface of the glass in these solutions. Glycerol reduces the ionic exchange of the glass with the solution, suggesting also a minor ability to bind polyphenols. However, it seems that this molecule does not remain grafted on the glass surface after functionalization. This result was confirmed by both Folin–Ciocalteu and fluorescence microscopy measurements, which highlighted a stronger presence of polyphenols on the samples CEL2 + BUDS ROSA and CEL2 + BYPRODUCT ROSA functionalized without the presence of glycerol in the solutions. Polyphenols are also present on the surface of the other samples but in a lower amount. The XPS analysis, according to the fluorescence microscopy images, showed the presence of a uniform layer of biomolecules on the surface of the samples, with much more agglomerates on CEL2 + BUDS ROSA and CEL2 + BYPRODUCT ROSA. In vitro bioactivity tests were performed in order to check whether the samples are still bioactive after the procedure of functionalization. From the FTIR analysis, it appears that the presence of the polyphenols from bud extracts not only preserves bioactivity but also enhances it, promoting an abundant deposition of hydroxyapatite. This result together with the maintenance of biomolecule antioxidant activity after grafting (demonstrated by the Folin–Ciocalteu test) demonstrates the possibility of effectively combining the bioactive glass properties with those of the *R. canina* bud extracts. This combination allows a local multifunctional action of the biomaterial for a physiological healing. The solution that showed the greatest potential is that obtained using the byproducts of primary extraction in glycerol macerate. The main potential of this solution is the possibility of promoting the transformation of a byproduct (residues from glycerol macerate production) in a high added value molecule (polyphenols) source through a simple process (conventional solvent extraction). These molecules could effectively be used for the preparation of multifunctional materials with a green approach and a sustainable use of resources. Future works will be devoted to biological investigation of the advantages of the functionalized glass on cells by in vitro tests.

## References

[ref1] Folashade O.; Omoregie H.; Ochogu P. Standardization of herbal medicines-A review. Int J Biodvers Conserv 2012, 4, 101–112. 10.5897/IJBC11.163.

[ref2] Okigbo R.; Anuagasi C.; Amadi J. E. Advances in selected medicinal and aromatic plants indigenous to Africa. J. Med. Plant Res. 2009, 3, 086–095. 10.5897/JMPR.9000046.

[ref3] Campanini E., Dizionario di fitoterapia e piante medicinali. Tecniche nuove: 2012**,** 952.

[ref4] Donno D.; Beccaro G. L.; Cerutti A.; Mellano M. G.; Bounous G. J. V. R.; Rao L. G. Bud Extracts as New Phytochemical Source for Herbal Preparations—Quality Control and Standardization by Analytical Fingerprint. 2015, 187–218, 10.5772/59759.

[ref5] Ahan N. Correlation of polyphenolic contents of indigenous medicinal plants with their bioactivities. UNIVERSITY OF AGRICULTURE, Doctoral dissertation, FAISALABAD PAKISTAN, 2011.

[ref6] Pharmaciens ONd Paris, Pharmacopée Française, Codex Medicamentarius Gallicus, Codex Français: Monographie, Préparations Homéopathiques. 1965.

[ref7] Lattanzio F.; Greco E.; Carretta D.; Cervellati R.; Govoni P.; Speroni E. In vivo anti-inflammatory effect of Rosa canina L. extract. J. Ethnopharmacol. 2011, 137, 880–885. 10.1016/j.jep.2011.07.006. 21771653

[ref8] Ieri F.; Innocenti M.; Possieri L.; Gallori S.; Mulinacci N. Phenolic composition of “bud extracts” of Ribes nigrum L., Rosa canina L. and Tilia tomentosa M. J. Pharm. Biomed. Anal. 2015, 115, 1–9. 10.1016/j.jpba.2015.06.004. 26135753

[ref9] Kharazmi A.; Winther K. Rose hip inhibits chemotaxis and chemiluminescence of human peripheral blood neutrophils in vitro and reduces certain inflammatory parameters in vivo. InflammoPharmacology 1999, 7, 377–386. 10.1007/s10787-999-0031-y. 17657440

[ref10] Orodan M.; Vodnar D. C.; Toiu A. M.; Pop C. E.; Vlase L.; Viorica I.; Arsene L. Phytochemical analysis, antimicrobial and antioxidant effect of some gemmotherapic remedies used in respiratory diseases. Farmacia 2016, 64, 224–230. http://www.revistafarmacia.ro/201602/art-11-OrodanVlase_Arsene_224-230.pdf.

[ref11] Chrubasik C.; Roufogalis B. D.; Müller-Ladner U.; Chrubasik S. A systematic review on the Rosa canina effect and efficacy profiles. Phytother 2008, 22, 725–733. 10.1002/ptr.2400. 18384191

[ref12] Schwager J.; Richard N.; Schoop R.; Wolfram S. A novel rose hip preparation with enhanced anti-inflammatory and chondroprotective effects. Mediators Inflammation 2014, 2014, 1 10.1155/2014/105710. PMC4211164 25371599

[ref13] Demir F.; Özcan M. Chemical and technological properties of rose (Rosa canina L.) fruits grown wild in Turkey. Food Eng 2001, 47, 333–336. 10.1016/S0260-8774(00)00129-1.

[ref14] Orhan N.; Aslan M.; Hosbas S.; Deliorman O. D. Antidiabetic effect and antioxidant potential of Rosa canina fruits. Phcog Mag 2009, 5, 309–315. 10.4103/0973-1296.58151.

[ref15] Ercişli S.; Eşitken A. Fruit characteristics of native rose hip (Rosa spp.) selections from the Erzurum province of Turkey. N Z J Crop Hortic Sci 2004, 32, 51–53. 10.1080/01140671.2004.9514279.

[ref16] Ercisli S. Chemical composition of fruits in some rose (Rosa spp.) species. Food Chem. 2007, 104, 1379–1384. 10.1016/j.foodchem.2007.01.053.

[ref17] Serteser A.; Kargioğlu M.; Gök V.; Bağci Y.; Özcan M. M.; Arslan D. Nutrition, Determination of antioxidant effects of some plant species wild growing in Turkey. Int. J. Food Sci. Nutr. 2008, 59, 643–651. 10.1080/09637480701602530. 19382350

[ref18] Quave C. L.; Plano L. R. W.; Pantuso T.; Bennett B. C. Effects of extracts from Italian medicinal plants on planktonic growth, biofilm formation and adherence of methicillin-resistant Staphylococcus aureus. J. Ethnopharmacol. 2008, 118, 418–428. 10.1016/j.jep.2008.05.005. 18556162 PMC2553885

[ref19] Puleo D.; Nanci A. Understanding and controlling the bone–implant interface. Biomaterials 1999, 20, 2311–2321. 10.1016/S0142-9612(99)00160-X. 10614937

[ref20] Hildebrand H.; Blanchemain N.; Mayer G.; Chai F.; Lefebvre M.; Boschin F. Surface coatings for biological activation and functionalization of medical devices. Surf. Coat. Technol. 2006, 200, 6318–6324. 10.1016/j.surfcoat.2005.11.086.

[ref21] Cazzola M.; Corazzari I.; Prenesti E.; Bertone E.; Vernè E.; Ferraris S. Bioactive glass coupling with natural polyphenols: Surface modification, bioactivity and anti-oxidant ability. Appl. Surf. Sci. 2016, 367, 237–248. 10.1016/j.apsusc.2016.01.138.

[ref22] Zhang X.; Ferraris S.; Prenesti E.; Verné E. Surface functionalization of bioactive glasses with natural molecules of biological significance, Part I: Gallic acid as model molecule. Appl. Surf. Sci. 2013, 287, 329–340. 10.1016/j.apsusc.2013.09.151.

[ref23] Zhang X.; Ferraris S.; Prenesti E.; Verné E. Surface functionalization of bioactive glasses with natural molecules of biological significance, part II: Grafting of polyphenols extracted from grape skin. Appl. Surf. Sci. 2013, 287, 341–348. 10.1016/j.apsusc.2013.09.152.

[ref24] Chen Q. Z.; Rezwan K.; Armitage D.; Nazhat S. N.; Boccaccini A. R. The surface functionalization of 45S5 Bioglass®-based glass-ceramic scaffolds and its impact on bioactivity. J. Mater. Sci. Mater. Med. 2006, 17, 979–987. 10.1007/s10856-006-0433-y. 17122908

[ref25] Schuhladen K.; Roether J. A.; Boccaccini A. R. Bioactive glasses meet phytotherapeutics: The potential of natural herbal medicines to extend the functionality of bioactive glasses. Biomaterials 2019, 217, 119288 10.1016/j.biomaterials.2019.119288. 31252243

[ref26] Kargozar S.; Kermani F.; Mollazadeh Beidokhti S.; Hamzehlou S.; Verné E.; Ferraris S.; Baino F. Functionalization and surface modifications of bioactive glasses (BGs): tailoring of the biological response working on the outermost surface layer. Materials 2019, 12, 3696 10.3390/ma12223696. PMC6888252 31717516

[ref27] Sayed Abdelgeliel A.; Ferraris S.; Cochis A.; Vitalini S.; Iriti M.; Mohammed H.; Kumar A.; Cazzola M.; Salem W. M.; Verné E. Surface functionalization of bioactive glasses with polyphenols from padina pavonica algae and in situ reduction of silver ions: physico-chemical characterization and biological response. Coatings. 2019, 9 ( (6), ), 394, 10.3390/coatings9060394.

[ref28] Cazzola M.; Vernè E.; Cochis A.; Sorrentino R.; Azzimonti B.; Prenesti E.; Rimondini L.; Ferraris S. Bioactive glasses functionalized with polyphenols: In vitro interactions with healthy and cancerous osteoblast cells. J. Mater. Sci. 2017, 52, 9211–9223. 10.1007/s10853-017-0872-5.

[ref29] Turrini F.; Donno D.; Boggia R.; Beccaro G. L.; Zunun P.; Leardi R.; Pittaluga A. M. An innovative green extraction and re-use strategy to valorize food supplement by-products: Castanea sativa bud preparations as case study. Food Research International 2019, 276–282. 10.1016/j.foodres.2018.12.018. 30599942

[ref30] Vitale-Brovarone C.; Verné E.; Robiglio L.; Martinasso G.; Canuto R. A.; Muzio G. Biocompatible glass–ceramic materials for bone substitution. J. Mater. Sci. 2008, 19, 471–478. 10.1007/s10856-006-0111-0. 17607523

[ref31] Verné E.; Vitale-Brovarone C.; Bui E.; Bianchi C. L.; Boccaccini A. R. Surface functionalization of bioactive glasses. J. Biomed. Mater. Res. 2009, 90A, 981–992. 10.1002/jbm.a.32153. 18655138

[ref32] Verné E.; Ferraris S.; Vitale-Brovarone C.; Spriano S.; Bianchi C. L.; Naldoni A.; Morra M.; Cassinelli C. Alkaline phosphatase grafting on bioactive glasses and glass ceramics. Acta Biomater. 2010, 6, 229–240. 10.1016/j.actbio.2009.06.025. 19540371

[ref33] Ferraris S.; Miola M.; Cochis A.; Azzimonti B.; Rimondini L.; Prenesti E.; Vernè E. In situ reduction of antibacterial silver ions to metallic silver nanoparticles on bioactive glasses functionalized with polyphenols. App Surf Sci 2017, 396, 461–470. 10.1016/j.apsusc.2016.10.177.

[ref34] Singleton V. L.; Rossi J. A. Viticulture, Colorimetry of total phenolics with phosphomolybdic-phosphotungstic acid reagents. Am J Enol Vitic 1965, 16, 144–158.

[ref35] Ferraris S.; Zhang X.; Prenesti E.; Corazzari I.; Turci F.; Tomatis M.; Vernè E. Gallic acid grafting to a ferrimagnetic bioactive glass-ceramic. J. Non-Cryst. Solids 2016, 432, 167–175. 10.1016/j.jnoncrysol.2015.05.023. 27694048

[ref36] Zandomeneghi M.; Carbonaro L.; Caffarata C. Fluorescence of vegetable oils: olive oils. 2005, 53 ( (3), ), 759–766, 10.1021/jf048742p. 15686431

[ref37] Aina V.; Magistris C.; Cerrato G.; Martra G.; Viscardi G.; Lusvardi G.; Malavasi G.; Menabue L. New formulation of functionalized bioactive gGlasses to be used as carriers for the development of pH-stimuli responsive biomaterials for bone diseases. Langmuir 2014, 30, 4703–4715. 10.1021/la5003989. 24701982

[ref38] Andersson Ö. H.; Kangasniemi I. Calcium phosphate formation at the surface of bioactive glass in vitro. J. Biomed. Mater. Res. 1991, 25, 1019–1030. 10.1002/jbm.820250808. 1918106

[ref39] Textor M.; Sittig C.; Frauchiger V.; Tosatti S.; Brunette D. M. , Properties and Biological Significance of Natural Oxide Films on Titanium and Its Alloys. In Titanium in Medicine: Material Science, Surface Science, Engineering, Biological Responses and Medical Applications , Springer Berlin Heidelberg: Berlin, Heidelberg, 2001**.** pp. 171–230, 10.1007/978-3-642-56486-4_7.

[ref40] Ferraz M. P.; Monteiro F. J.; Santos J. D. CaO-P_2_O_5_ glass hydroxyapatite double-layer plasma-sprayed coating: In vitro bioactivity evaluation. J. Biomed. Mater. Res. 1999, 45, 376–383. 10.1002/(SICI)1097-4636(19990615)45:4<376::AID-JBM13>3.0.CO;2-S. 10321711

[ref41] Ferraris S.; Perero S.; Vernè E.; Battistella E.; Rimondini L.; Ferraris M. Surface functionalization of Ag-nanoclusters–silica composite films for biosensing. Mater. Chem. Phys. 2011, 130, 1307–1316. 10.1016/j.matchemphys.2011.09.019.

[ref42] Vernè E.; Ferraris S.; Vitale-Brovarone C.; Cochis A.; Rimondini L. Bioactive glass functionalized with alkaline phosphatase stimulates bone extracellular matrix deposition and calcification in vitro. Appl. Surf. Sci. 2014, 313, 372–381. 10.1016/j.apsusc.2014.06.001.

[ref43] Morra M.; Cassinelli C.; Bruzzone G.; Carpi A.; Santi G. D.; Giardino R.; Fini M. Surface chemistry effects of topographic modification of titanium dental implant surfaces: 1. Surface analysis. Int J Oral Maxillofac Implants 2003, 18 (). 12608667

[ref44] Biesinger M., X-ray photoelectron spectroscopy reference pages. January: 2012**.**

